# Scientific Evidence Supporting the Beneficial Effects of Isoflavones on Human Health

**DOI:** 10.3390/nu12123853

**Published:** 2020-12-17

**Authors:** Saioa Gómez-Zorita, Maitane González-Arceo, Alfredo Fernández-Quintela, Itziar Eseberri, Jenifer Trepiana, María Puy Portillo

**Affiliations:** 1 Nutrition and Obesity Group, Department of Pharmacy and Food Science, University of the Basque Country (UPV/EHU) and Lucio Lascaray Research Institute, 01006 Vitoria, Spain; saioa.gomez@ehu.eus (S.G.-Z.); maitane.gonzalez@ehu.eus (M.G.-A.); alfredo.fernandez@ehu.eus (A.F.-Q.); mariapuy.portillo@ehu.eus (M.P.P.); 2 CIBEROBN Physiopathology of Obesity and Nutrition, Institute of Health Carlos III, 01006 Vitoria, Spain; 3 Bioaraba Health Research Institute, 01002 Vitoria, Spain

**Keywords:** isoflavones, flavonoids, phytoestrogens, soy, bone health, cardiovascular risk, cancer, menopausal symptoms

## Abstract

Isoflavones are phenolic compounds with a chemical structure similar to that of estradiol. They are present in several vegetables, mainly in legumes such as soy, white and red clover, alfalfa and beans. The most significant food source of isoflavones in humans is soy-derived products. Isoflavones could be used as an alternative therapy for pathologies dependent on hormonal disorders such as breast and prostate cancer, cardiovascular diseases, as well as to minimize menopausal symptoms. According to the results gathered in the present review, it can be stated that there is scientific evidence showing the beneficial effect of isoflavones on bone health and thus in the prevention and treatment of osteoporosis on postmenopausal women, although the results do not seem entirely conclusive as there are discrepancies among the studies, probably related to their experimental designs. For this reason, the results should be interpreted with caution, and more randomized clinical trials are required. By contrast, it seems that soy isoflavones do not lead to a meaningful protective effect on cardiovascular risk. Regarding cancer, scientific evidence suggests that isoflavones could be useful in reducing the risk of suffering some types of cancer, such as breast and endometrial cancer, but further studies are needed to confirm these results. Finally, isoflavones could be useful in reducing hot flushes associated with menopause. However, a limitation in this field is that there is still a great heterogeneity among studies. Lastly, with regard to isoflavone consumption safety, it seems that they are safe and that the most common adverse effect is mild and occurs at the gastrointestinal level.

## 1. Introduction

**Figure 1:**
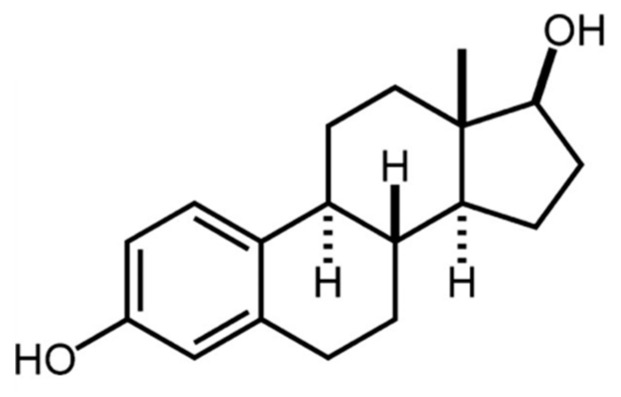
Chemical structure of 17β-estradiol. Modified from Wang et al., 2006 [4].

Phenolic compounds are secondary metabolites, which are produced by plants as a defense mechanism against infection, water stress, cold stress, ultraviolet radiation and high visible light, among others [1,2]. Phenolic compounds, characterized by the presence of a hydroxyl group attached to at least one aromatic ring, can be classified as flavonoids and non-flavonoids. Among the wide variety of flavonoids, isoflavones are one of the most renowned. Due to its structural similarity to the estrogen-like compound 17β-estradiol (Figure 1), they are referred to as phytoestrogens. Consequently, isoflavones can have estrogenic or anti-estrogenic effects [3].

Isoflavones have received attention due to their putative healthy properties. In this sense, it has been described that both isoflavones and other estrogenic molecules could mediate their beneficial effects due to two different mechanisms: the classical estrogen receptor (ER)-mediated signaling pathway and the activation of intracellular pathways such as protein tyrosine kinase, phospholipase C and mitogen-activated protein kinase (MAPK) [5,6]. In the aforementioned mechanism, it has been reported that isoflavones bind to both ERα and ERβ, although they present a higher affinity towards ERβ receptors [7]. Moreover, even though the binding affinity of isoflavones to the ER receptors is less than that of 17β-estradiol, they can also act as estrogenic compounds when the endogenous estradiol is not available [8]. Thus, its consumption, according to epidemiological and clinical studies, has been postulated to be related to a decrease in the risk of different diseases. The aim of the present review is to gather the scientific evidence existing nowadays on the main beneficial effects of isoflavones on health: bone health, cardiovascular risk, cancer and menopausal symptoms. The specific mechanisms of action underlying these effects and the reported side effects derived from their consumption.

## 2. Oral Intake and Occurrence

Isoflavones are found in several vegetables, mainly in legumes (*Fabaceae* family) such as soy, white and red clover, alfalfa and beans [9,10]. The most significant food source of isoflavones in humans is soy-derived products, soybeans, soy flour, soy flakes, soy beverages and fermented soy products such as miso and tempeh, among others [9]. Although smaller in quantity, isoflavones are also present in chickpeas, nuts, fruits and vegetables [11].

**Figure 2:**
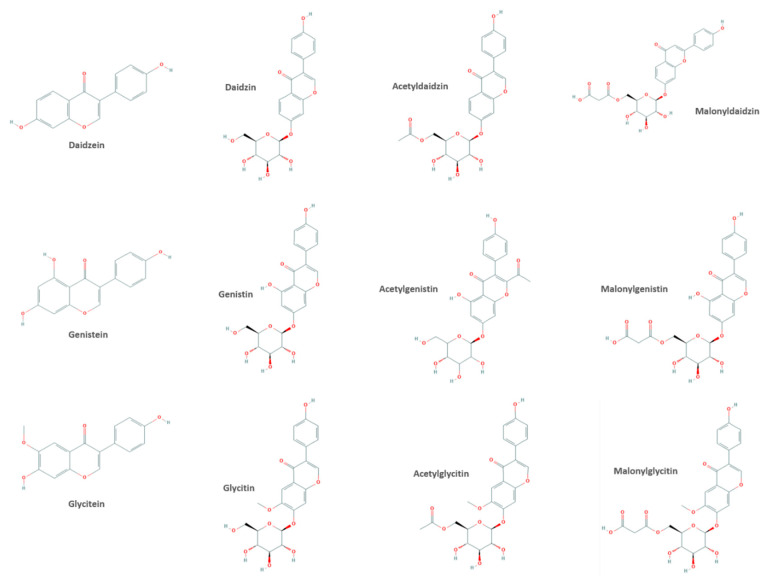
The four chemical forms of the three most abundant isoflavones.

The main isoflavones found in foodstuffs are daidzein, genistein and glycitein, as well as biochanin A and formononetin [9,12]. Some authors label equol as an isoflavone; however, since it is a daidzein-derived bacterial metabolite and not a naturally occurring isoflavone, this classification is not correct [10]. Nevertheless, it is important to consider this metabolite since it is key in the biological activity of isoflavones [13]. As with other phenolic compounds, isoflavones can be presented as free forms (aglycones) or conjugated with carbohydrates (glycosides). The latter is the most common form in foodstuffs, with the exception of fermented soy products, such as miso and tempeh. In miso and tempeh, the aglycone form is the most abundant one [9]. In soybeans, the three most abundant isoflavones are present in four chemical forms: aglycone, glycoside, acetylglycoside and malonylglucoside (Table 1 and Figure 2). The aglycone and conjugate forms of genistein represent up to 60% of total isoflavones, this being up to 30% in the case of daidzein. The main forms of conjugates in soybeans are the malonyl derivatives [14].

With regard to isoflavone content in foodstuffs, it has been observed that in soybeans, the amount of these phenolic compounds ranges from 1.2 to 4.2 mg per g on dry weight, whereas in red clover, the amount varies between 10 and 25 mg per g on dry weight [15,16]. Even though red clover has a greater amount of isoflavones, it is not an edible plant, and it is consumed as a food complement. Thus, soybean can be considered as the best food source. Isoflavone content in soy-derived products is usually lower than in soybeans [17]. Importantly, the soy variety, environmental conditions of the culture and the processing of soybeans notably influence their isoflavone content [18]. Furthermore, isoflavones are associated with proteins; thus, alcohol extraction and processing diminish isoflavone content in foodstuffs such as fermented soybean, tofu or soy beverages. By contrast, the total isoflavone amount is high in soy flour, protein isolate or edamame [19].

Focusing on the overall intake of isoflavones in humans, several studies have reported varied results. In general terms, it has been observed that in Asian countries where there is high consumption of soy and its derived foodstuffs, isoflavone intake is high, ranging from 15 to 60 mg/day [20,21], whereas, in western countries, it is notably lower, around 1–2 mg/day [22,23,24].

## 3. Bioavailability

As previously mentioned, soybeans and their derived foodstuffs are the main isoflavone sources in the human diet, at least if we do not consider red clover extract supplements, which are being widely used by menopausal women as “nutraceutical therapy” [13]. For this reason, the bioavailability of isoflavones has been extensively characterized in animal models and humans [25,26,27].

Isoflavone glycosides have to be hydrolyzed to the aglycone form prior to its absorption by passive diffusion in the upper small intestine [28]. The enzymes responsible for the hydrolysis of isoflavone glycosides are glucosidases, which can be produced by the intestinal mucosa or the microbiota [29]. The enzyme lactase-phlorizin hydrolase (LPH) deglycosylases phenolic compounds in the intestinal lumen, and after that, the aglycone form enters the epithelial cells by passive diffusion. Moreover, sodium-dependent glucose transporter 1 (SGLT1) allows phenolic compound-glycosylates to directly enter into epithelial cells, where they are hydrolyzed by cytosolic glucosidases [30,31]. Interestingly, in a study conducted by Tamura et al., the authors observed that in individuals without the LPH enzyme, the concentration of isoflavones and derived metabolites was similar to that found in individuals without an enzyme deficiency. This finding suggests that bacterial deglycosylation contributes significantly to isoflavone absorption [32]. Daidzein, genistein and glycitein occur naturally in the aglycone form, and thus their absorption is faster than that of others.

It has been described that after the oral intake of isoflavones, their peak plasma concentration in humans occurs at around seven hours [33]. As in the case of other phenolic compounds, genistein and daidzein undergo phase II xenobiotic metabolism, mainly glucuronidation and sulfation reactions at 4’and/or 7’ positions of the isoflavone ring [34,35]. Produced metabolites appear in plasma at variable concentrations and can enter enterohepatic circulation [28]. In a study reported by Ko et al., the authors quantified the plasma concentrations of daidzein, genistein, glycitein and equol in Korean men and women [36]. After the consumption of around 17 mg of isoflavones per day, the median plasma concentrations of genistein, glycitein, daidzein and equol were 245.3 ng/mL, 9.8 ng/mL, 86.8 ng/mL and 12.7 ng/mL, respectively.

**Figure 3:**
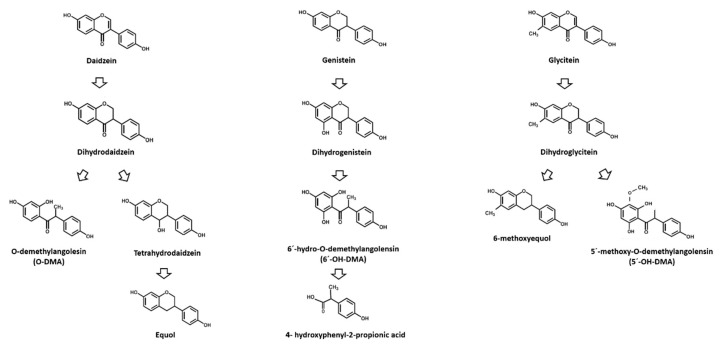
Bacterial metabolites of isoflavones forming in the gut. Modified from Mace et al. and Rossi et al. [12,42].

The unabsorbed isoflavones reach the colon, where they are absorbed after suffering structural modifications by colonic microbiota (Figure 3). Daidzein is first converted into dihydrodaidzein, which is the precursor of both equol and O-demethylangolensin (O-DMA) [37]. Equol has been considered as the most interesting colonic metabolite due to its beneficial biological activity, which differs from that of its precursor, daidzein [13,38,39]. In addition, genistein and glycitein are transformed into dihydrogenistein and dihydroglycitein, respectively, before being further converted into other metabolites. Nevertheless, the metabolism of formononetin and biochanin A, the main isoflavones in red clover, has been less studied due to their limited presence in foodstuffs. Anywise, it has been observed that both isoflavones are demethylated by microbiota, formononetin to daidzein and biochanin A to genistein. After this, each metabolite undergoes its own metabolic pathway [40]. In addition, it is important to highlight that interindividual differences in gut bacterial populations are responsible for the diverse effects of isoflavones in humans. While equol production seems to be similar in animals, it is very variable in humans [41], and therefore, beneficial effects referred to equol have been observed only in individuals with a specific microbiota composition [38].

Isoflavone excretion occurs mainly through urine and feces, primarily in the conjugate forms, and up to 95% takes place in 24 h [43]. In addition, several studies have observed that the urine excretion of daidzein metabolites is notably higher than that of genistein metabolites [44,45,46]. In a study conducted by Karr et al., the authors analyzed the urinary excretion of genistein, daidzein, equol and O-DMA in fourteen young men and women [47]. They observed that it was dose-dependent at low to moderate soybean consumption.

## 4. Biological Activity

Isoflavones could be used as an alternative therapy for pathologies dependent on hormonal disorders such as breast and prostate cancer, cardiovascular diseases, as well as to minimize menopausal symptoms. For the development of this section, the meta-analyses and systematic reviews published between 2015 and 2020 were taken into account.

### 4.1. Bone Health Maintenance

During menopause, there is a loss of bone density that can cause osteoporosis. In this sense, soy isoflavones have been proposed as beneficial because, in theory, they may contribute to the maintenance of good bone health (mass, mineral density and bone structure) in women who are at this stage in their life. Regarding this topic, three systematic reviews and three meta-analyses were analyzed (Table 2).

In the systematic review reported by Perna et al. (2016), nine studies addressed to menopausal women and focused on bone health were analyzed [52]. The authors indicated that no consensus was found regarding the protective effects of soy isoflavones (20–80 mg) and equol (10 mg) on bone resorption. Abdi et al. (2016) published another systematic review of 23 randomized controlled trials to determine the effect of soy isoflavone extracts on bone mineral density in postmenopausal women [51]. The conclusion was that isoflavones exerted little influence over bone mineral density and thus over bone health during menopause, although not all the studies observed the same effect. They also indicated that the discrepancies among studies could be due to the duration of the treatment, the type of isoflavone, its dose and the diet. Regarding the effect of specific isoflavones, they suggested that genistein, alone or in combination with daidzein, improved bone density and bone turnover in women after menopause. However, the authors stated that they could not reach definitive conclusions due to, among other reasons, the lack of inclusion in the review of randomized controlled clinical trials focused on the treatment of osteoporosis in early menopause. In a more recent systematic review, including two meta-analyses, as well as one multicenter and randomized controlled trial, Chen and his team concluded that isoflavones reduced lumbar spine bone mineral density loss [53]. It is important to point out that the spine has a high proportion of trabecular bone and that this is probably the reason why the spine is thought to be more sensitive to isoflavones.

With regard to the published meta-analysis, the studies included were restricted to randomized controlled trials. The work reported by Lambert et al. (2017) included 26 trials with 2652 participants [48]. It supports the evidence that isoflavones can moderately attenuate bone resorption in women with low estrogen levels, primarily at the level of the lumbar spine and the femoral neck. They also note that the effects of isoflavones are greater when administered as aglycones. This fact was not taken into account in previous meta-analyses, and thus it may justify the discrepancies amongst them. In this line, another meta-analysis, which included 52 controlled trials and 5313 patients, shows that soy isoflavones have beneficial effects on bone mineral density in the femur neck, lumbar spine, and hip, regardless of body weight or ethnicity [49]. The improvement was greater in treatments lasting more than one year and in subjects with normal weight, probably because subjects with excessive body weight have a lesser risk of bone loss. In contrast, the effects on bone resorption biomarkers were more favorable in overweight or obese women than in women with a healthy weight. Sansai et al. (2020) carried out their meta-analysis of 63 controlled trials, including 6427 postmenopausal women and concluded that isoflavones improve bone density at the lumbar spine, femoral neck and the distal radius in menopausal women [50]. These positive effects were associated with 54 mg/day of genistein and 600 mg/day of synthetic isoflavone ipriflavone.

In view of these results, we can conclude that there is scientific evidence that supports the beneficial effect of isoflavones on bone health and thus in the prevention and treatment of osteoporosis in postmenopausal women. However, the results do not seem entirely conclusive, for there are discrepancies among the studies, probably related to their experimental designs. For this reason, the results should be interpreted with caution, and more randomized clinical trials are required.

**Figure 4:**
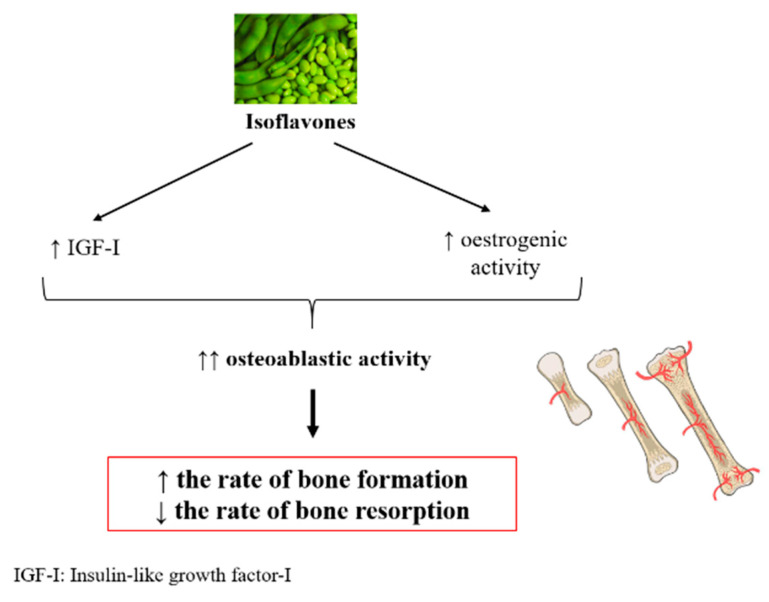
Potential mechanism of action of isoflavones on bone metabolism.

Although the mechanisms of action of isoflavones are not completely understood, it seems that isoflavones not only reduce the rate of bone resorption but also increase the rate of bone formation. The enhanced bone formation is due to the stimulation of osteoblastic activity mainly through (a) the activation of estrogen receptors because they bind to nuclear estrogen receptors and exhibit estrogenic activity due to its similarity to 17β-estradiol, and (b) the promotion of insulin-like growth factor-I (IGF-I) production [54,55] (Figure 4).

### 4.2. Cardiovascular Risk

In some Asian countries, isoflavone intake, usually derived from soy consumption, can be associated with a lower prevalence of cardiovascular diseases (CVDs). In this revision, three systematic reviews and five meta-analyses were included to explore the association of isoflavones and markers related to CVDs (Table 3).

In their systematic revision, which included 1307 menopausal (*n* = 139) and post-menopausal (*n* = 1268) women, Perna et al. (2016) suggested that a daily intake of soy isoflavones, ranging from 20 to 100 mg/day, reduced total cholesterol and triglyceride plasma concentrations, as well as some markers of oxidative stress (nitric oxide and malonaldehyde), thus reducing cardiovascular risk [52]. Further, Chalvon-Demersay et al. aimed to compare the effects of animal and plant-sourced proteins on lipemia and blood pressure [60]. In this systematic review, 123 studies were included, with a total number of 516,330 participants. The authors found, in a small number of studies (17 studies including 337 healthy, diabetic or hypercholesterolemic individuals), that a soy protein-based diet (rich in isoflavones) as compared to animal protein-based diets (meat, milk, casein, whey) resulted in reduced total cholesterol or LDL-cholesterol concentrations, increased HDL cholesterol concentrations, improved LDL:HDL cholesterol ratio, and decreased triglycerides. Interestingly, soy protein isolate (void of isoflavones through alcoholic extraction during the protein solation phase) did not show these effects. Regarding blood pressure, inconsistent results were reported in three further interventional studies (*n* = 406 healthy, postmenopausal or obese participants aged 50–79 years) either in diastolic or systolic blood pressure, after a supplementation with soy-protein.

In another systematic review of three observational studies (including 68,748 participants), low evidence of isoflavones was shown in the prevention of cardiovascular diseases [61]. The authors concluded that evidence for the role of isoflavones and soy products in the prevention of cardiovascular diseases was scarce and inconclusive.

Two meta-analyses, which covered prospective cohort studies, showed that soy isoflavone consumption was not associated significantly with mortality linked to cardiovascular events [56,62]. Indeed, Kim and Je (2017) indicated that a high intake of flavonoids is associated with a reduced risk of mortality from cardiovascular diseases in men and women [56]. However, when a subgroup analysis by a class of flavonoids was carried out, they concluded that these inverse associations were significant for all categories of flavonoids except for isoflavones and flavonols.

Additionally, Yan and co-workers, in their meta-analysis with a total of ten prospective cohorts and seven case-control studies (including 508,841 participants and 17,269 cardiovascular disease events), observed that the consumption of soy foods, but not that of isoflavones, was associated with a lower risk of total cardiovascular disease (including coronary heart disease and stroke) [57]. This fact suggests that other components, such as fiber in the soy foods, may account for these negative associations. In like manner, a meta-analysis by Simental-Mendía et al. (2018) showed no effect of soy isoflavones on plasma concentrations of lipoprotein a (Lpa), a low-density lipoprotein associated with increased cardiovascular risk due to its pro-thrombotic and atherogenic properties [63].

Lastly, Abshirini et al. (2020) revised the literature to find randomized controlled trials that evaluated the impact of soy protein supplementation (isoflavone intake ranging from 49.3 to 118 mg/day) on endothelial function parameters in postmenopausal women (*n* = 802) [58]. Apparently, soy isoflavones modified adhesion molecules by binding to vascular endothelium and mimicking the effect of a modulator of estrogen receptors [64]. Furthermore, soy protein has been linked to an improvement in nitric oxide production and contributes to increased arterial compliance [65]. However, in the meta-analysis, a minor enhancement in flow-mediated dilation after soy protein supplementation was found. On the contrary, a systematic review and meta-analysis reported by Man et al. (2020) revealed that supplementation of soy isoflavones had a positive effect in reducing arterial stiffness, also known as the loss of arterial elasticity [59]. The physical stiffening of arteries has major health implications for its connection to various adverse cardiovascular and other health outcomes such as coronary heart disease, stroke, hypertension or heart failure [66]. However, the authors acknowledged several limitations to their study, such as the small sample size in the meta-analysis (middle-aged 276 women and 209 men), and recognized that a large number of subjects as well as a lengthier intervention are needed to investigate whether supplementation of soy isoflavones actually improves these outcomes.

Taken as a whole, all these results suggest that soy isoflavones do not lead to a meaningful protective effect on cardiovascular risk. It follows that additional high-quality, large-scale randomized controlled trials are needed.

As far as the mechanisms of action involved in the decrease in triglyceride concentration are concerned, it has been reported that hepatic lipase and/or lipoprotein lipase activity may be increased as a result of isoflavone presence. Indeed, some authors speculate that hepatic and lipoprotein lipase activity may have been altered after variable isoflavone intake from soybean, although no experimental data were provided [67]. Since hepatic lipase and lipoprotein lipase hydrolyze triglycerides into their constituents glycerol and fatty acids, their activation results in the delivery of the fatty acids to tissues such as muscle and adipose tissue.

### 4.3. Cancer

Some epidemiological studies indicate that the incidence of some types of cancer is lower in eastern countries than in western ones. This fact does not seem to be influenced by genetics since migrating from eastern to western countries appears to cause the loss of this protective effect. In this section, 16 meta-analyses have been revised: Nachvack et al. (2019) conducted a meta-analysis where 23 prospective studies with a total of 330,826 participants were included. The authors observed that soy isoflavone consumption was inversely associated with cancer deaths. Moreover, a higher intake of soy isoflavones was associated with a lower risk of mortality from gastric, colorectal, and lung cancers. Indeed, they reported that an increase of 10 mg/day of soy isoflavones consumption was associated with a 7% lower risk of cancer mortality [62].

Regarding breast cancer (Table 4), the same increase in soy isoflavone intake (10 mg/day) was related to a 9% lower risk of breast cancer mortality. However, it is important to point out that the beneficial effects of soy isoflavone consumption have been exhibited by women with estrogen receptor-negative breast cancer, but not by women with receptor-positive breast cancer, who present a better prognosis [62]. In this sense, Micek et al. (2020) carried out a meta-analysis with 15 cohort studies with the aim of exploring the association between isoflavone intake (<62.7 mg/day) and breast cancer mortality and its recurrence [68]. The authors found a significant inverse association between isoflavone intake and both overall mortality and breast cancer recurrence. These two associations were significant for postmenopausal participants. In addition, Qiu et al. (2018) analyzed 12 studies with 37,275 women with breast cancer and reported that soy isoflavone consumption at a pre-diagnosis stage might have a small effect on the survival of postmenopausal women with breast cancer [69]. However, the authors pointed out several limitations of this study, such as the important source of heterogeneity in their study. In fact, the number of isoflavones present was very variable among the different soy food types. Therefore, it is necessary to support these results with additional studies. Future research in this field should quantify isoflavones as accurately as possible, analyzing larger cohorts and lengthening the follow-up stage. Moreover, the number of publications grouping women according to pre- or post-diagnosis intake criteria is small.

Another meta-analysis, conducted by Zhao et al. (2019), included 16 prospective cohort studies involving 11,169 breast cancer cases and 648,913 participants [70]. The authors studied all possible correlations between isoflavone consumption and the risk of breast cancer. While a moderate intake of soy isoflavones did not significantly affect breast cancer risk, a significant reduction was shown when the intake was high. However, some limitations were found in these prospective studies, such as the small sample size of the cohorts and the unspecific definition of high, moderate and low isoflavone intake. Moreover, Rienks et al. (2017) conducted a meta-analysis with ten observational studies to assess the association between the isoflavone compounds daidzein, genistein and equol, and breast cancer [61]. The authors indicated that high concentrations of daidzein and genistein (no specific values concerning these concentrations were available) were related to 34% and 28% reduction in breast cancer risk, respectively, whereas no association with equol was found. In conclusion, this study reported some evidence that suggests the beneficial role that daidzein and genistein play in preventing breast cancer risk. Furthermore, among the four studies of breast cancer recurrence, three studies found that isoflavone intake decreased the risk of breast cancer recurrence, although further studies are required to confirm this finding [82].

Endometrial cancer (Table 4), as well as breast cancer, is an estrogen-dependent cancer type related to high circulating estradiol concentrations. In this line, three meta-analyses were conducted to study isoflavone consumption and endometrial cancer risk. Zhong et al. (2018) revised 13 epidemiologic studies involving 178,947 participants: 7067 cases and 171,880 controls; among them, three were prospective cohort studies, and ten were population-based case-control studies [71]. The authors reported that isoflavone intake from soy products and legumes was associated with a 19% reduction in endometrial cancer risk. However, the magnitude of risk reduction in Asian women was slightly larger than in non-Asian women (22% vs. 18%). Diversely, Liu et al. (2016) analyzed the effect of oral isoflavone supplementation on endometrial thickness in pre- and post-menopausal women, which is a biomarker for the proliferative effects of estrogens associated with an increased endometrial risk [72]. For this purpose, 23 studies with 2167 patients were included in this meta-analysis, which showed that a daily dose of more than 54 mg could decrease the endometrial thickness in North American women, whereas the contrary effect was observed in the Asian sample. The authors suggested that this difference could be due to different genetic backgrounds and dietary patterns among Asian and Western populations. Grosso et al. (2017) carried out a meta-analysis of 143 case-control studies, where isoflavone intake was associated with endometrial, ovarian, and breast cancers [73]. Regarding endometrial cancer, three prospective and five case-control studies were analyzed, which suggested a lower endometrial cancer risk associated with isoflavone consumption. However, more studies are required to provide stronger evidence.

Hua et al. (2016) conducted a meta-analysis aimed at ovarian cancer, which affects women exclusively [74]. In this study, five prospective cohort studies and seven case-control studies, with 6275 cases and 393,776 controls, were included. The results indicated that the isoflavone intake decreased the ovarian cancer risk by 33%. Therefore, the authors concluded that the intake of dietary isoflavones paid a protective role against ovarian cancer.

With regard to prostate cancer, it has been proposed that it might be affected by isoflavone consumption due to its effect on hormone metabolism. Four meta-analyses have been reported to address this issue. Pérez-Cornago et al. (2018) published a meta-analysis of seven prospective cohort studies (two studies from Japan with 241 cases and 503 controls, and five studies from Europe with 2828 cases and 5593 controls) [75]. In Japanese men, pre-diagnostic circulating levels of genistein, daidzein and equol did not significantly affect prostate cancer risk. In the same line, in European men, isoflavone concentrations did not affect the risk of developing prostate cancer. In the meta-analysis reported by Applegate et al. (2018), 30 articles were included for analysis; 15 of them were case-control studies, eight cohort studies and seven nested case-control studies [76]. This meta-analysis included 266,699 participants (21,612 patients with prostate cancer among them). The authors reported that neither soy food intake nor circulating isoflavones were associated with a reduced risk of prostate cancer. However, the intake of specific isoflavones, genistein and daidzein, was indeed significantly associated with a reduced risk of this type of cancer. The authors suggested that further studies grouping subjects by isoflavone intake or by the circulating isoflavone levels might enhance this association. Rienks et al. (2017) also analyzed eight studies of prostate cancer, and they found an association between isoflavone intake and prostate cancer risk [61]. A significant 19% reduction of the risk was found at high concentrations of daidzein, but not at genistein or equol concentrations. Lastly, Zhang et al. (2017) conducted a meta-analysis with 21 case-control studies and two cohort studies, with a total number of 11,346 cases and 140,177 controls [77]. They observed that daidzein and genistein intakes were associated with a significant reduction of prostate cancer risk. Nevertheless, this association was not shown when equol or isoflavones were consumed. Moreover, the authors suggested that ethnicity could have had influenced this association, but studies with larger sample sizes are needed to confirm this hypothesis.

Due to the high prevalence of colorectal cancer, three meta-analyses addressing this issue are included in the present review. He et al. (2016) conducted a meta-analysis of 18 studies involving 16,917 colorectal cancer cases in 559,486 participants [78]. The results showed that a higher regular intake of foods rich in isoflavones might potentially decrease colorectal cancer incidence. Moreover, this association was more prominent among postmenopausal women than premenopausal women. However, the authors suggested that more prospective cohort studies are needed to further investigate this association and that the quantity of isoflavone intake should be accurately measured because flavonoid contents in food can vary significantly. In the same line, Yu et al. (2016) published a meta-analysis of 13 case-control and four prospective cohort studies, where the results revealed that soy isoflavone consumption reduced the risk of developing colorectal cancer by 23% [79]. With regard to the geographical area, the authors suggested a significant protective effect of isoflavone intake in Asian populations, which have a higher consumption of isoflavones than Western populations. For their meta-analysis, Jiang et al. (2016) included 17 studies (nine case-control studies and eight cohort studies) [80]. The authors found a statistical inverse association between isoflavone consumption and colorectal cancer risk in case-control studies, although not in cohort studies. Furthermore, the dose-response analysis revealed an 8% reduction in colorectal neoplasm risk for every 20 mg/day increase in isoflavone intake in Asian populations, and for every 0.1 mg/day increase in Western populations. The difference for isoflavones required to find an effect can be due to variations in the amount of isoflavone intake between both populations. Thus, whereas Asian populations have an average isoflavone consumption of >30 mg/day, Western populations consume <1 mg/day [83].

Lastly, the association between dietary isoflavone intake and the risk of developing gastric cancer has also been analyzed. You et al. (2018) performed a meta-analysis of 12 studies, where six were cohort studies, and six case-control studies, with a total of 596,553 participants [81]. The results showed no significant association between isoflavone consumption and gastric cancer risk, with the highest (0.6–75.5 mg/day) *versus* the lowest (0.01–20.1 mg/day) categories of dietary isoflavone intake. In this sense, the authors suggested that the composition of the gut microflora may influence isoflavone absorption and metabolism and could affect the production of isoflavone metabolites, which, in turn, could mediate their biological activity.

Overall, evidence regarding the use of isoflavones in cancer prevention suggests that they may be useful in reducing the risk of suffering from some types of cancer, such as breast and endometrial cancer, two of the types for which the association between isoflavone intake and cancer risk has been studied more in-depth. However, all the authors agree that further studies are necessary so as to confirm these results. Indeed, the dose levels of dietary isoflavones require a better definition, and the measurements of isoflavone consumption should be provided in quantifiable terms.

**Figure 5:**
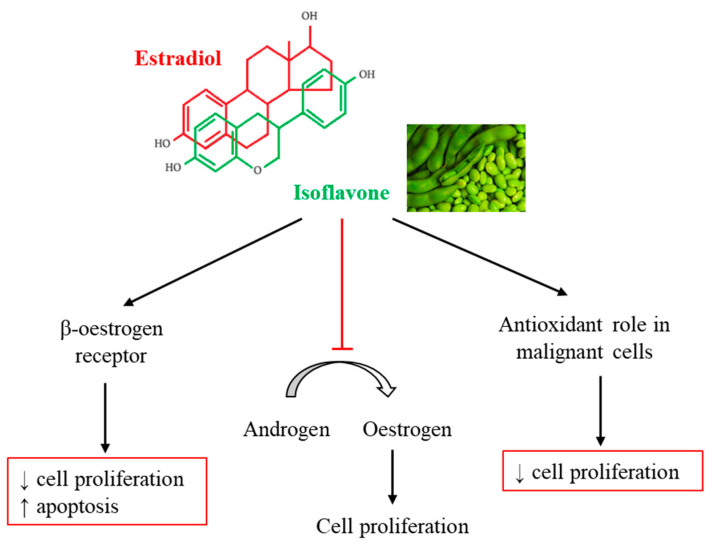
Possible mechanism of action of isoflavones for cancer prevention.

With regard to the mechanisms by which isoflavones act to reduce cancer risk, it has been suggested that the effects are due to the similarity of the isoflavone molecule to estradiol. This chemical feature confers estrogenic or antiestrogenic effects to isoflavones, depending on the cell type and the binding to α- or β-estrogen receptors. In this regard, it has been reported that isoflavones have a higher affinity to the β-estrogen receptor [7], which, contrary to the α-estrogen receptor, inhibits cell proliferation and stimulates apoptosis [84]. Furthermore, isoflavones are able to inhibit aromatase activity, the enzyme that converts androgen to estrogen. It is also important to mention that isoflavones could act as potential anticancer compounds due to their antioxidant role in malignant cell proliferation and differentiation (Figure 5).

### 4.4. Menopausal Symptoms

Menopause is characterized by a decrease in estrogen levels and is often accompanied by a range of symptoms. Among these, vasomotor symptoms comprising hot flushes and night sweats are the most common and bothersome, which, in turn, have a negative impact on women’s quality of life. Hormone replacement therapy has been proven to be effective in reducing vasomotor symptoms, although isoflavones have gained popularity as an alternative treatment to hormone replacement therapy to relieve menopausal symptoms. This is mainly due to their potential adverse effects, which range from increased coronary heart disease to stroke and cancer [53,85]. Concerning this issue, six meta-analyses and two systematic reviews are included in the present review (Table 5).

The six meta-analyses show a slight improvement in the frequency and intensity of hot flushes. In the meta-analysis reported by Chen et al. (2015), the authors examined the efficacy of phytoestrogens in reducing hot flushes [86]. The meta-analysis included 15 studies in which the number of participants ranged from 30 to 252. The mean age of women ranged from 49 to 58.3 and 48 to 60.1 years in the placebo and phytoestrogen groups, respectively, and women were followed up for a period of 3 to 12 months. Isoflavones were the phytoestrogens used in most of the studies; therefore, the meta-analysis only included outcomes regarding isoflavones, while outcomes with other phytoestrogens were not included. Ten studies reported a significant reduction in hot flush frequency in the phytoestrogen group when compared with the placebo group. Moreover, five studies reported side-effect data, and after the analysis, no significant differences were observed between the two groups.

Similarly, Li et al. (2015) concluded that soy isoflavones have both a slight and slow effect in attenuating menopausal hot flushes [87]. A total of 16 studies were included in their meta-analysis with a range of 24 to 236 subjects. Study duration ranged from four weeks to two years, with a median of 12 weeks. The dose of isoflavones ranged from 30 to 200 mg/day. Despite the positive effect of isoflavones, the authors emphasized that the effects took a long time to appear. Estradiol, which has proven efficacy on hot flushes, needs 3.09 weeks to achieve half of its peak activity, while isoflavones require 13.4 weeks, suggesting that 12 weeks of treatment is not enough for soy isoflavones to exert a beneficial effect. The same results were reported in another meta-analysis published by the same authors a year later (Li et al., 2016), when they compared the efficacy of several non-hormonal drugs on hot flushes, including selective serotonin reuptake inhibitors (SSRIs), serotonin–norepinephrine reuptake inhibitors (SNRIs), gabapentin, clonidine and soy isoflavones [88]. Thirty-nine studies were included with a sample size of 200 women per trial, in which the intervention period ranged from two to 96 weeks (median 12 weeks). As in the previous study, the onset of soy isoflavones was very slow in comparison with other non-hormonal treatments. Specifically, the time to achieve half of the efficacy was 11.6 weeks for soy isoflavones, while for the rest of the compounds, the onset time was near 0 weeks. They also explained that the differences between isoflavones and the rest of the drugs might be because the latter act by modulating central neurotransmitters and, as a consequence, by regulating the central thermoregulatory centers in the hypothalamus. In contrast, it seems that isoflavones do not act on central neurotransmitters directly. This difference could explain the slow effect of isoflavones in alleviating hot flushes.

Daily et al. (2019) studied whether equol supplementation could benefit equol nonproducer subjects in lowering the incidence and severity of hot flushes [85]. Equol is produced from the isoflavone daidzein by intestinal bacteria, mainly in the large intestine. Their meta-analysis included five studies with a total of 728 menopausal women between 50.5 and 58.5 years old and concluded that women who are not able to produce equol could benefit from equol supplementation. On the contrary, women who were already equol producers did not obtain any additional benefit from supplements of equol or isoflavones.

Another meta-analysis explored the effects of several pharmacological and non-pharmacological treatments in the relief of vasomotor symptoms, taking into account not only hot flushes but also night sweats [89]. Non-pharmacological treatments included isoflavones, which appeared to be better than placebo in the relief of vasomotor symptoms, although not significantly better than the treatment with estradiol and progesterone. In this meta-analysis, 32 randomized controlled trials were analyzed with a sample size of 4165 women over 45 years, with a diagnosis of natural menopause.

Franco et al. (2016) also studied whether there was an association between different types of interventions and the reduction in hot flushes and night sweats [90]. They performed a separated meta-analysis for each of the interventions, including dietary soy isoflavones (including four studies), supplements and extracts of soy isoflavones (including six studies) and red clover isoflavones (including seven studies). They found that the use of dietary isoflavones or supplements and extracts was associated with an improvement in hot flushes, but not with a reduction in the frequency of night sweats. In contrast, red clover was not associated with changes in the hot flush frequency. Only one study examined the effects of red clover on night sweats, and a significant association was found, which showed a decreased frequency in night sweats.

The two systematic reviews conclude that isoflavones have an important role in reducing hot flushes [52,53], although some observations need to be pointed out. Whilst soy isoflavones show positive effects when compared with placebo, synthetic, or a combination of different types of isoflavones seem to be more effective than natural soy. When a group receiving hormone replacement therapy was included in the study, significant differences between the effects of hormone replacement therapy and soy were found, being the effect of the former superior to that of isoflavones. Two of the studies also revealed that women who were able to produce equol or who had received equol supplementation could have had a greater benefit from isoflavones [53]. Perna et al. (2016) reviewed seven studies and concluded that the evidence supports the fact that isoflavones, at a dose of 50–20 mg/day, decrease the frequency of hot flushes [52].

**Figure 6:**
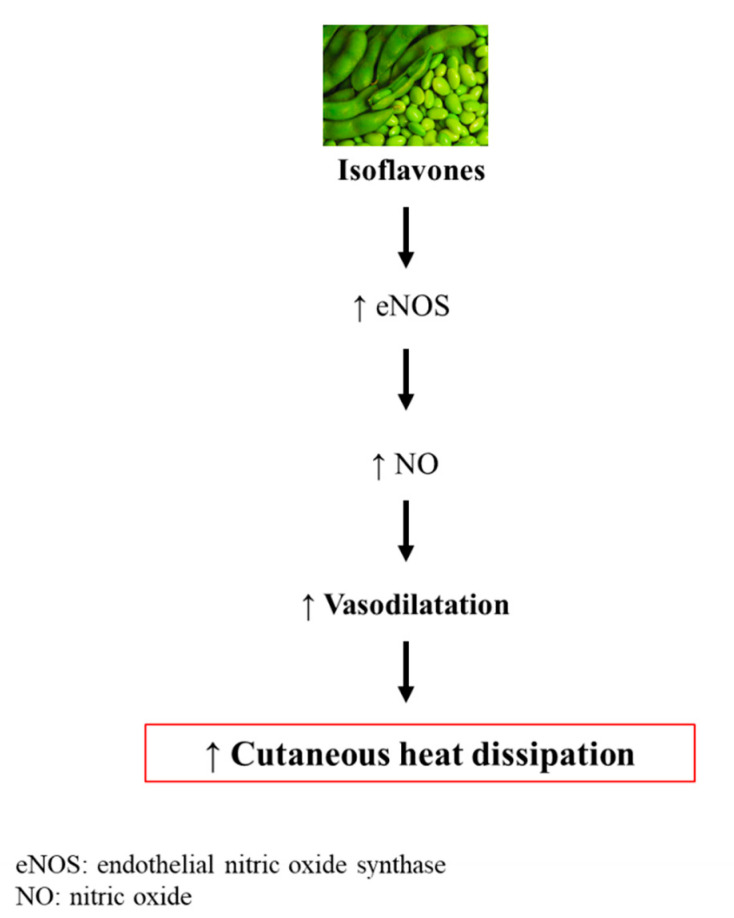
Possible mechanism of action of isoflavones for menopausal symptoms.

In general, evidence regarding the use of isoflavones on vasomotor symptoms suggests that they could be useful in reducing hot flushes. However, there is still a great heterogeneity among studies, which makes the current data inconclusive. Regarding the mechanisms by which isoflavones act to alleviate vasomotor symptoms, is has been proposed that they bind to estrogen receptors and activate endothelial nitric oxide synthase (eNOS) transcription, leading to eNOS synthesis and nitric oxide production, the main mediator of vasodilatation, which allows cutaneous heat dissipation [91] (Figure 6).

## 5. Side Effects and Safety

Potential health adverse effects in the intake of isoflavones have been postulated due to their estrogenic activity, especially in relation to breast cancer. However, it is important to mention that this connection has been established based on pre-clinical studies, whereas data derived from clinical trials have not demonstrated such association [92]. In fact, in 2015, in relation to the possible adverse effects on the mammary gland, uterus and thyroid, the European Food Safety Agency (EFSA) offered a scientific opinion with regards to the assessment of potential increased risk in peri- and postmenopausal women who took food supplements that contained isolated isoflavones. After an extensive bibliographical review, the expert panel concluded that the results of observational studies do not support the hypothesis of an increased risk of breast cancer after isoflavone-rich food supplement intake [93]. Moreover, an increase in thyroid hormone levels after isoflavone intake by food supplements has not been observed, and no side effects were confirmed with regard to the endometrial thickness or histopathological changes in the uterus. Thus, the EFSA scientific committee’s opinion agreed that isoflavone intake was safe, at least in the doses that the analyzed studies have used. In any case, it is important to bear in mind that a great number of expert scientists and doctors think that breast cancer survivors should not increase their isoflavone intake [94], although others consider that their consumption is safe and beneficial [95]. In general terms, caution should be exercised with the use of high doses of isoflavones in women with a family history of breast cancer [49].

Finally, it is important to mention that despite the fact that potential isoflavone adverse effects have been widely studied, little is known about the side effect of the bacterial metabolite equol. As it has been stated in the Bioavailability section, microbiota composition in humans makes equol production very variable, which yields high interindividual differences. Reproductive and developmental toxicity studies have stated that 1 g and 2 g of equol per day is safe [96], although further studies are required [92].

## 6. Concluding Remarks

In view of the results gathered in the present review, we can conclude that there is scientific evidence that reveals the beneficial effect of isoflavones on bone health and thus in the prevention and treatment of osteoporosis in postmenopausal women. Nevertheless, the results do not seem entirely conclusive, as there are discrepancies among the studies included in the systematic reviews and in the meta-analyses, probably related to their experimental designs. For this reason, the results should be interpreted with caution, and more randomized clinical trials are imperative. By contrast, it seems that soy isoflavones do not lead to a meaningful protective effect on cardiovascular risk. Regarding cancer, scientific evidence suggests that isoflavones could be useful in reducing the risk of suffering from some types of cancer, such as breast and endometrial cancer, although further studies are needed to confirm these results. In the end, isoflavones could be useful in reducing hot flushes associated with menopause. However, a limitation in this field is that there is still a great heterogeneity among studies. Altogether, these results show that further studies are needed in order to find stronger conclusions.

Considering the isoflavone chemical structure, potential health adverse effects of these compounds related to their estrogenic activity have been postulated, despite the scientific opinion offered by EFSA indicating that isoflavone intake was safe.

## Figures and Tables

**Table 1:** Presence of four chemical forms of the three isoflavones found in soybeans.

Isoflavone Content and Chemical Forms in Soybeans	
Aglycones	Daidzein, genistein, glycitein
Glycosides	Daidzin, genistin, glycitin
Acetylglycosides	Acetyldaidzin, acetylgenistin, acetylglycitin
Malonylglycosides	Malonyldaidzin, malonylgenistin, malonylglycitin

**Table 2:** Effects of isoflavones in bone health maintenance.

Authors	Number of Studies Included	Type of Studies Included	Number of Participants and Gender/Age/Characteristics	Compound and Doses	Observed Effects
Meta-analysis					
Lambert et al., 2017 [48]	26	Randomized Clinical trials	2652 estrogen-deficient women	Isoflavones (different forms) Intervention period: ≥3 months	Moderate attenuation of bone loss, primarily at the level of the lumbar spine and the femoral neck
Akhlaghi et al., 2019 [49]	52	Controlled trials	5313 patients	Soy isoflavones 40–300 mg/day Intervention period: 1 month–3 years	Prevention of osteoporosis-related bone loss in any weight status or treatment duration
Sansai et al., 2020 [50]	63	Controlled trials	6427 postmenopausal women	Isoflavones (different forms) Intervention period: 1–36 months	Isoflavone interventions, genistein (54 mg/day) and ipriflavone (600 mg/day) in particular hold great promise in the prevention and treatment of bone mineral density
**Systematic reviews**					
Abdi et al., 2016 [51]	23	Clinical trials	3494 participants	Isoflavones Intervention duration: 7 weeks–3 years	Probably they have beneficial effects on bone health in menopausal women but there are controversial reports about changes in bone mineral density
Perna et al., 2016 [52]	9	Clinical trials	1379 menopausal and postmenopausal women	Soy isoflavones (20–80 mg) and equol (10 mg)	May be protective in osteoporosis
Chen et al., 2019 [53]	3	1Meta-analysis 1Systematic review and 1clinical trial	3663 menopausal and postmenopausal women	Soy isoflavones	Attenuation of lumbar spine bone mineral density

**Table 3:** Effects of isoflavones in cardiovascular disease-related markers.

Authors	Number of Studies Included	Type of Studies Included	Number of Participants and Gender/Age/Characteristics	Compound and Doses	Observed Effects
Meta-analysis					
Kim and Je, 2017 [56]	13	Prospective studies	338,541 participants Age ranging from 40–84 years Follow-up period from 4 to 28 years	Intake data not provided	No association with mortality from CVD
Yan et al., 2017 [57]	17	Prospective cohort and case-control studies	508,841 participants, 17,269 with CVD events (stroke, coronary heart disease, ischemic stroke) Follow-up period from 6.3 to 16 years	Isoflavones 0.025–53.6 mg/day	No associations between soy isoflavones consumption and risk of cardiovascular disease, stroke, and coronary heart disease
Abshirini et al., 2020 [58]	5	Randomized clinical trials	548 participants (272 case and 276 controls) Age not available	Isoflavones 49.3–118 mg/day Intervention period: 1–12 months	Non-significant change in flow-mediated dilation (parameter of endothelial function)
Man et al., 2020 [59]	8	Various designs (double-blind, placebo-controlled, parallel design, crossover design)	485 participants (276 women and 209 men) Age ranging from 35–75 years	Isoflavones 80–118 mg/day and 10 mg/day of S-equol Intervention period: 1 day–12 weeks	Positive effect of soy isoflavones on arterial stiffness
**Systematic reviews**					
Perna et al., 2016 [52]	12	Randomized clinical trials	139–1268 menopausal and postmenopausal women	Isoflavones 20 to 100 mg/day Intervention period: 8 weeks–2 years	Reduction in total cholesterol and triglyceride plasma concentrations Reduction in nitric oxide and malonaldehyde
Chalvon-Demersay et al., 2017 [60]	17 3	Randomized clinical trials; nutritional intervention	337 healthy, diabetic or hypercholesterolemic individuals Age: 18–74 years 406 healthy, postmenopausal or obese participants Age: 50–79 years	Isoflavones 3 to 102 mg/day Intervention period: 4–208 weeks Isoflavones 60 to 135 mg/day Intervention period: 3–12 months	Reduction in total cholesterol and LDL cholesterol Changes in systolic or diastolic blood pressure (increase and decrease, depending on the study)
Rienks et al., 2017 [61]	3	Prospective studies	68,748 individuals Age: 40–70 years	Follow-up period: up to 10 years	Decreased risk of acute coronary syndrome or coronary heart disease No association with ischemic stroke

CVD: cardiovascular disease, LDL: low-density lipoprotein.

**Table 4:** Effects of isoflavones in different cancer types.

Authors	Number of Studies Included (Meta-Analysis)	Type of Studies Included	Number of Participants and Gender/Age Characteristics	Compound and Doses	Observed Effects
Nachvack et al., 2019 [62]	23	Prospective study	330,826 (12 studies in both genders and 11 in women)	Soy/soy products (10 mg/day)	Inverse association with cancer deaths. 7% lower risk of gastric, colorectal, and lung cancer mortality
**Breast cancer**					
Nachvack et al., 2019 [62]	23	Prospective study	330,826 (12 studies in both genders and 11 in women)	Soy/soy products (10 mg/day)	9% lower risk of estrogen receptor-negative breast cancer mortality
Micek et al., 2020 [68]	15	Cohort study	49,659	Isoflavone intake (0.0036–62.7 mg/day)	Inverse association between isoflavone intake and both overall mortality and breast cancer recurrence
Qiu et al., 2018 [69]	12	Prospective cohort study	37,275 women	Isoflavones (the amount varies greatly among different soy foods)	Pre-diagnosis, soy isoflavone consumption has a poor effect on survival of postmenopausal women
Zhao et al., 2019 [70]	16	Prospective cohort study	648,913 (11,169 breast cancer cases)	High dietary intake of soy foods (dose non-defined)	Significant reduction of breast cancer risk
Rienks et al., 2017 [61]	10	Case-control study	Sample sizes (from 100 to 15,688 participants)	Daidzein, genistein, and equol (dose non-defined)	Daidzein (34%) and genistein (28%) were associated with a lower risk of breast cancer
**Endometrial cancer**					
Zhong et al., 2016 [71]	13	Prospective cohort study (3) Case-control study (10)	178,947 (7067 cases and 171,880 controls)	Soy products (0.05–130 g/day) and total isoflavones (0.28 to 63 mg/day).	19% reduction in endometrial cancer risk. Higher reduction in Asian women
Liu et al., 2016 [72]	23	Randomized controlled trials	2167	More than 54 mg isoflavone/day	Reduction of the endometrial thickness in North American women (for 0.23 mm). Opposite effect in Asian women
Grosso et al., 2017 [73]	8	Prospective studies (3) Case-control studies (5)	Non-defined	Isoflavone consumption (>45 mg/day among the Asian population, and >1 mg/day among the non-Asian population)	Potential reduction risk associated with isoflavone consumption
**Ovarian cancer**					
Hua et al., 2016 [74]	12	Prospective cohort study (5) Case-control study (7)	6275 cases and 393,776 controls	Isoflavone intake (0.01–41 mg/day)	33% reduction in ovarian cancer risk
**Prostate cancer**					
Pérez-Cornago et al., 2018 [75]	7	Cohort prospective study (2 studies from Japan and 5 studies from Europe)	241 cases and 503 controls (from Japanese studies), and 2828 cases and 5593 controls (from European studies) 60–69 years-old	Circulating isoflavone concentrations (nmol/L): Daidzein (Japanese: 115–166; European: 2.84–3.96) Genistein (Japanese: 277–454; European: 4.84–5.97), and equol (Japanese: 10.3–24; European: 0.25–0.65)	Genistein, daidzein and equol did not affect prostate cancer risk in both Japanese and European men
Applegate et al., 2018 [76]	30	Case-control study (15) Cohort study (8) Nested case-control study (7)	266,699 (21,612 patients with prostate cancer)	Soy foods (<90 mg/day)	Isoflavones were not associated with a reduction of prostate cancer risk Metabolites (genistein and daidzein) consumption was associated with a reduction of prostate cancer risk
Rienks et al., 2017 [61]	8	Case-control study	Sample sizes (10–15,688 participants)	Daidzein, genistein, and equol (dose not defined)	19% reduction in prostate cancer risk was found at high concentrations of daidzein, but not with genistein or equol
Zhang et al., 2017 [77]	23	Case-control study (21) Cohort study (2)	Participants: 11,346 cases and 140,177 controls	Daidzein, genistein, and equol (dose not defined)	Daidzein and genistein intakes were associated with a reduction of prostate cancer risk (no effect with equol)
**Colorectal cancer**					
He et al., 2016 [78]	18	Case-control study (9) Cohort study (9)	559,486 (among them 16,917 colorectal cancer cases)	Foods rich in isoflavones (dose not defined)	Reduction of colorectal cancer risk. This association is stronger among postmenopausal women than premenopausal women
Yu et al., 2016 [79]	17	Case-control study (13) Prospective cohort study (4)	272,296 participants	Soy foods (30 mg/day–170 g/day) and isoflavones (0.014–60 mg/day)	23% reduction in colorectal cancer risk. Potential protective effect in the Asian population (their consumption is higher than in the Western population)
Jiang et al., 2016 [80]	17	Case-control study (9) Cohort study (8)	317,599 participants	Isoflavones (0.025–74 mg/day).	Inverse association between isoflavone consumption and colorectal cancer risk in case-control studies, but not in cohort studies. 8% reduction in colorectal neoplasm risk for every 20 mg/day increase in isoflavone intake (Asian population) and for every 0.1 mg/day increase (Western population)
**Gastric cancer**					
You et al., 2018 [81]	12	Cohort study (6) Case-control study (6)	596,553 participants	Isoflavones (high dose: 0.6–75.5 mg/day; and low dose: 0.01–20.1 mg/day).	No association between isoflavone consumption and gastric cancer risk with the highest versus the lowest categories of dietary isoflavone intake

**Table 5:** Effects of isoflavones in menopausal symptoms.

Authors	Number of Studies Included	Type of Studies Included	Number of Participants and Gender/Age/Characteristics	Compound and Doses	Observed Effects
Meta-analysis					
Chen et al., 2015 [86]	15	RCT	30–252 perimenopausal or postmenopausal women/report (1753 in total) 49–58.3 years (placebo group) 48–60.1 years (phytoestrogen group)	Isoflavones 5–100 mg/day Intervention period: 3–12 months	Reduction of hot flush frequency (vs. placebo)
Li et al., 2015 [87]	16	RCT	24–236 women/report (median 90) 40–65 years	Soy isoflavones 30–200 mg/day Intervention period: 4 weeks–2 years (median 12 weeks)	Slight and slow attenuation of hot flushes (vs. estradiol)
Li et al., 2016 [88]	39	RCT	24–620 women/report (median 200) Age not available	SSRIs/SNRIs: 7.5–200 mg/day Gabapentin: 300–1800 mg/day Clonidine: 0.1–0.4 mg/day Soy isoflavones: 30–200 mg/day Intervention period: 2–96 weeks (average 12 weeks)	Slight and slow attenuation of hot flushes (vs. non-hormonal drugs)
Daily et al., 2019 [85]	5	RCT	728 menopausal women (total subjects) 50.5–58.8 years (mean)	Soy isoflavones: 33–200 mg/day and 6 g soy extract/day Equol: 10 mg/day Intervention period: not available	Equol or isoflavone in equol-producers more effective than placebo
Sarri et al., 2017 [89]	32	RCT	4165 menopausal women (total subjects) 45+ years	Isoflavones and black cohosh (Doses not available)	Reduction of VSM (hot flushes and night sweats) compared to placebo No beneficial effect (vs. pharmacological treatment)
Franco et al., 2016 [90]	17	RCT	30–252 women/trial 40–69 years	Dietary soy isoflavones: 42–90 mg/day Supplements and extracts of soy isoflavones: 10–100 mg/day Red clover: 40–160 mg/day Intervention period: 12–48 weeks	Reduction of hot flush frequency by means of dietary isoflavones and supplements) Reduction of night sweat frequency by red clover
**Systematic reviews**					
Chen et al., 2019 [86]	15	RCT (9) Prospective study (2) Systematic review (2) Randomized crossover trial (1) Meta-analysis (2)	51–403 menopausal and postmenopausal women	Soy (soy nut, soy protein, soy extracts) Natural isoflavones Synthetic isoflavones	Beneficial effects of isoflavones (vs. placebo) Synthetic or combination of isoflavones more effective than natural soy HRT more effective than soy or its extracts Isoflavone in equol-producers or equol supplementation more effective than placebo.
Perna et al., 2016 [52]	7	RCT	40–403 menopausal and postmenopausal women	Isoflavones 50–120 mg/day Intervention period: 8 weeks–2 years	Reduction of hot flush frequency

HRT: hormone replacement therapy, RCT: randomized controlled trial, VSM: vasomotor symptoms.
